# An Atlas of Surra in Spain: A Tool to Support Epidemiological Investigations and Disease Control

**DOI:** 10.3390/ani14020243

**Published:** 2024-01-12

**Authors:** Adrián Melián Henríquez, María Teresa Tejedor-Junco, Margarita González-Martín, Manuel Morales Doreste, Sergio Martín Martel, Massimo Paone, Giuliano Cecchi, Juan Alberto Corbera

**Affiliations:** 1 Instituto Universitario de Investigaciones Biomédicas y Sanitarias (IUIBS), Universidad de Las Palmas de Gran Canaria (ULPGC), 35016 Las Palmas, Spain; 2 Departmento de Ciencias Clínicas, Universidad de Las Palmas de Gran Canaria (ULPGC), Paseo Blas Cabrera Felipe “Físico”, 17, Las Palmas de Gran Canaria, 35016 Las Palmas, Spain; 3 Hospital Clínico Veterinario-Universidad de Las Palmas de Gran Canaria (HCV-ULPGC), Campus Universitario de Arucas, 35413 Las Palmas, Spain; 4 Animal Production and Health Division, Food and Agriculture Organization of the United Nations (FAO), 00153 Rome, Italy

**Keywords:** Spain, Canary Islands, atlas, African animal trypanosomosis, *Trypanosoma* spp., *T. evansi*, database, epidemiology

## Abstract

**Simple Summary:**

Trypanosomosis is a widespread issue in animals, resulting in significant economic losses, particularly in Africa. In Spain, only one pathogenic species, *Trypanosoma evansi,* has been identified. Since its first detection in a dromedary camel in the Canary Islands in 1997, cases of the disease, known as Surra, have continued to be diagnosed, leading to various studies and control efforts. Due to the lack of a comprehensive database consolidating the most relevant data on *T. evansi* in Spain, the development of a national atlas was deemed necessary, with a focus on the Canary Islands. To develop the atlas, a repository was created that compiled data and documents from 1997 to 2022. Information from different sources, including georeferenced locations and blood test results, was extracted and integrated into a comprehensive database. The analysis of 31 sources provided 99 georeferenced locations and 12,433 animal samples. Out of these samples, 601 were found to be positive for *T. evansi*, mostly from dromedaries. The Card Agglutination Test for *T. evansi* (CATT/*T. evansi*) was the most commonly used diagnostic method, showing a higher prevalence for all tested animal species. The positive cases were primarily concentrated in the Canary Islands, specifically in the eastern islands, with a few isolated cases in the province of Alicante in the Iberian Peninsula. This atlas serves as a comprehensive overview of the history and prevalence of Surra in Spain and provides a valuable tool for future control initiatives and research. However, further studies are still needed to investigate potential hosts other than camelids and their associated transmission vectors.

**Abstract:**

Trypanosomosis is a global animal issue, causing significant economic losses, particularly in Africa. In Spain, only one pathogenic species, *Trypanosoma evansi*, has been identified so far. It was first detected in a dromedary camel in the Canary Islands in 1997. Since then, numerous cases of the disease, known as Surra, have been diagnosed, prompting various studies and efforts in control and surveillance. Given the lack of a comprehensive database that consolidates the most relevant data in this area, the development of a national atlas, with a focus on the Canary Islands, to incorporate all available information on *T. evansi* in Spain became a necessity. For the development of the atlas, a repository was constructed, encompassing a range of datasets and documents spanning from 1997 to 2022. Information from each source, and in particular georeferenced locations and results of blood tests on animals, were extracted and integrated into a comprehensive database. A total of 31 sources were analysed, providing a total of 99 georeferenced locations and 12,433 animal samples. Out of these samples, 601 (mostly from dromedaries) were found to be positive for *T. evansi*. The Card Agglutination Test for *T. evansi* (CATT/*T. evansi*), a serological test, was the most commonly used diagnostic method, and it showed a higher prevalence for all tested animal species. Positive cases were mainly concentrated in the Canary Islands, specifically in the eastern islands, with isolated cases found in the province of Alicante (Iberian Peninsula). This atlas provides an overview of the history and occurrence of Surra in Spain, and it represents a valuable tool for future control initiatives and for research. Still, the need for more studies remains, especially for further testing of potential hosts other than camelids and for the examination of their potential transmission vectors.

## 1. Introduction

Trypanosomes are flagellated protozoa that belong to the family *Trypanosomatidae* and the genus *Trypanosoma*, encompassing a wide range of haemoparasitic protozoa that primarily affect vertebrates [1,2,3,4,5]. Several species, such as *Trypanosoma brucei*, *T. vivax*, *T. congolense*, *T. evansi* and *T. equiperdum*, are able to cause disease in domestic mammals, resulting in a substantial socio-economic impact and decreased animal productivity globally [6,7,8,9]. Livestock can also serve as a reservoir for human-infective trypanosomes, such as *T. brucei rhodesiense* and possibly *T. brucei gambiense*, significantly contributing to the transmission of sleeping sickness in humans [8,10,11,12,13]. Atypical infections of *T. evansi* in humans have also been reported [14,15,16].

The occurrence of *Trypanosoma* species in Europe has, up until now, solely been associated with the travel or immigration history of human patients or the introduction of infected animals [4,5,17,18]. However, there is limited information available on the epidemiological situation of trypanosomes that naturally infect mammals in Europe [4,7,19,20].

With the exception of *T. equiperdum*, which is sexually transmitted, these parasites are mainly transmitted by haematophagous insects. In Africa, these are mainly *Diptera* belonging to the *Glossina* genus, commonly known as tsetse flies, and part of the parasites’ life cycle occurs within these insects. However, one pathogenic species, *T. evansi*, is unique in that it does not undergo any development or replication within the tsetse flies [5,8,20,21]. Therefore, the transmission of the parasite is mechanically mediated, allowing it to extend its vector range to species from the *Stomoxys*, *Tabanus* and *Hippoboscidae* genera, as well as some species of bats (e.g., *Desmodus rotundus*) in certain parts of South America [1,22,23,24]. Additionally, other forms of transmission, such as horizontal (direct mucosal contact with parasitised secretions), vertical (transplacental), or iatrogenic (reusing or poorly cleaning material), have also been reported [1,3,25].

*T. evansi* is hypothesised to have originated from the species *T. brucei*, which is native to Africa. This species diverged as a result of the loss of the maxicircle kinetoplast DNA (kDNA), which likely restricted its biological cycle within different *Glossina* species. *T. evansi* expanded its range through animal transportation, such as dromedaries or equids from Africa [26]. Indeed, South America was reached through equids brought by Spanish conquistadors [2,27]. Reviews continue to reveal the expanding geographic distribution of *T. evansi*, which was originally confined to north-western, central, and eastern Africa but now extends to South America, Europe and Asia. Currently, *T. evansi* has been documented in 17 African nations, 7 South American nations, and 4 European nations [1,2]. Furthermore, recent investigations reveal that cases of Surra have been documented in 20 Asian nations [28,29].

Griffith Evans identified this pathogenic trypanosome in mammals in 1880 [1,2]. It can cause “Surra” disease in equids, bovids, canids, and humans, although humans may not develop any apparent symptoms [1,27,30,31]. Atypical infections in humans have been reported in Sri Lanka, India, Egypt, and Thailand [32]. However, it is widely accepted that the dromedary camel is the primary host of this parasite [1,33].

In the 1990s, Surra was first reported in Spain when it was detected in a dromedary camel in the Canary Islands [34]. The hypothesis that *T. evansi* was introduced to the islands from abroad is supported by the historically imported dromedaries from North African regions such as Morocco and Mauritania for agricultural and transport-related activities [35]. Following the discovery of *T. evansi* in the Canary Islands, several epidemiological studies were conducted to assess the prevalence of the disease in the local dromedary population [36].

Collaboration between the Institute of Tropical Medicine (Antwerp, Belgium) and the University of Las Palmas de Gran Canaria (ULPGC) was undertaken to examine a large part of the dromedary population on the islands (1012 dromedaries). From these, 745 animals (73.6%) were tested in 1999 with the Card Agglutination Test for *T. evansi* (CATT/*T. evansi*) and 36 (4.8%) resulted positive. Seven samples also tested positive on parasitological tests, mainly Giemsa-stained blood smears and micro-Haematocrit Centrifugation Technique (m-HCT) [7,36,37,38,39]. Subsequently, in the 2000s, an antibody Ab-ELISA (Enzyme-Linked ImmunoSorbent Assay) seroprevalence study was conducted on 444 samples of dromedaries from the islands of Fuerteventura, Lanzarote, Tenerife and Gran Canaria. The results showed a prevalence of 10% in Gran Canaria, 9.7% in Lanzarote, 7.5% in Tenerife and 7.8% in Fuerteventura [37]. Treatment of positive animals with melarsomine (Cymelarsan^®^, Merial, France) resulted in seronegative results on all tested islands except in Gran Canaria. In a study conducted on the latter island, which included around 200 animals, 5% of the sampled population tested positive in both serological and parasitological tests. In 2002, numerous abortions in dromedaries were associated with the parasite, which was identified through Giemsa-stained blood smears and m-HCT and treated with melarsomine. Changes in the biochemical parameters of the affected animals were further studied, yielding results described by Gutierrez et al. [28,29].

In 2008, *T. evansi* was detected in a camel and horse farm in the province of Alicante, mainland Spain, following the importation of a dromedary from the Canary Islands six months earlier. This animal presented clinical symptoms, including weight loss, weakness, and anaemia. After treating the dromedary with melarsomine, samples were taken from the other animals on the farm. As a result, 18 animals (16 camels and 2 horses) tested positive for CATT/*T. evansi*, with 12 of them (all camels) also testing positive through a parasitological test. Consequently, the affected animals were isolated and received the same treatment, effectively controlling the outbreak and eradicating the disease [17,40,41]. Similarly, in the department of Aveyron, France, another outbreak of *T. evansi* occurred in dromedaries due to the importation of dromedaries from the Canary Islands [42].

In 2014, there was another outbreak of the disease in the same location in Gran Canaria, where eradication had not been fully achieved previously. Several hypotheses were suggested to explain why the disease persisted, such as ineffective treatment of the animals or the presence of a reservoir in the affected area. To investigate this, the prevalence of the disease in domestic ruminants and equids was assessed, as well as the potential involvement of Diptera, such as *Stomoxys* spp. and rodents in its transmission [43,44,45]. Consequently, in 2017, the Department of Agriculture, Livestock and Fisheries of the Government of the Canary Islands established a monitoring and control system. This involved a yearly sampling of the entire dromedary population on the islands and testing for CATT/*T. evansi* and Polymerase Chain Reaction (PCR) of those animals positive to CATT/*T. evansi*. Positive animals were isolated and, in some cases, euthanised to prevent further transmission. The results of this programme were satisfactory, with only four animals testing positive serologically for *T. evansi* and no positive cases observed via PCR as of March 2022 [36].

In light of the various outbreaks of the disease reported in animals exported from the Canary Islands, there is a potential risk of spreading the disease to other territories. In order to better manage the existing and future information on Surra occurrence in Spain and gain insight into the epidemiological history of the disease and the current situation in the Canary Islands, the creation of a centralised database/information system was deemed necessary. To achieve this, a national ‘atlas’ was developed to compile and organise all available documents and data detailing the trypanosomosis caused by *T. evansi* since its initial detection in Spain.

The atlas was developed by the University of Las Palmas de Gran Canaria (ULPGC) in collaboration with the Department of Agriculture, Livestock and Fisheries of the Government of the Canary Islands. The government provided datasets collected for the purpose of controlling the incidence of this disease in dromedaries. Methodological guidance and supportive training were provided by the Food and Agriculture Organization of the United Nations (FAO). The tool was developed in the framework of the COMBAT project (Controlling and Progressively Minimizing the Burden of Animal Trypanosomosis) [46], which directly involves 21 institutions across different countries in Africa and Europe.

## 2. Materials and Methods

The atlas of Surra in Spain was developed by broadly following the methods developed by the FAO for the continental atlas of African Animal Trypanosomosis (AAT) and tsetse flies [47]. This approach has already been adapted and implemented by veterinary authorities in various African countries, resulting in the creation of several national atlases of AAT and tsetse occurrence [48,49,50,51,52,53]. These national databases are regarded as crucial tools for evidence-based planning of field interventions in the framework of the “progressive control pathway” for AAT [54].

For the Surra atlas in Spain, data were collected from multiple sources, mainly reports of investigations from different livestock farms. In these investigations, blood was sampled from various animal species, including dromedaries, goats, sheep, cows, horses, and donkeys. The primary purposes of these investigations were the targeting of veterinary treatment and academic research on the disease’s epidemiology, as well as ensuring its control and monitoring since its initial detection in Gran Canaria in the 1990s.

For diagnosis, several parasitological and serological methods were utilised, including CATT/*T. evansi* (Institute of Tropical Medicine, Belgium), ELISA [55], PCR [56], Woo technique [39], lymph node aspirate (LNA) [57], blood smears and MIT (Mice Inoculation Test) [58].

Animal samplings were conducted in different ways, with the initial ones being driven by clinical suspicion. Subsequently, a random and representative approach was adopted for certain studies to facilitate research, followed by an effort to cover a majority of the animal population (particularly dromedaries) through sources derived from control and monitoring efforts.

### 2.1. Data Sources and Development of the Atlas

In 2021, the process of acquiring documents and datasets related to Surra in Spain began. Each acquired document and dataset was added to a digital repository, and then, the most relevant information was extracted from the sources and included in a database. The data used for the development of this atlas were generated over a period of 25 years, from 1997 to 2022. They consist of 14 scientific publications, 7 conference proceedings, 1 PhD thesis and 4 documents of animal samples linked to the aforementioned scientific publications. Additionally, the atlas includes five unpublished datasets collected by the Department of Agriculture, Livestock and Fisheries of the Government of the Canary Islands. These datasets were gathered as part of their disease control efforts, including a comprehensive dromedary census on the islands conducted since 2018 [36]. All these sources cover specific regions of the Canary Islands, mostly the eastern islands, as well as a particular area of the Iberian Peninsula where Surra was diagnosed.

These documents were mainly obtained in PDF and Microsoft Excel formats, but older documents were initially only available in hard copy, so they had to be scanned and digitised. Sources were mostly identified by the main author’s surname and the date of publication or production. During this process, some papers were discarded, primarily those that detailed experimental inoculations of *T. evansi* in goats, which were performed to assess various diagnostic approaches and treatments.

The geographical coordinates of various sampling locations were obtained by utilising various applications, such as Google Earth and the Spatial Data Infrastructure of the Canary Islands (IDE Canarias). Localisation was completed at the level of the surveyed farm, and this information was included in the atlas as “location”. Subsequently, maps indicating the presence or absence of Surra in different target regions of Spain were generated and visualised via ArcGIS^®^ 10.8.2 software by Environmental Systems Research Institute, Inc. (ESRI), Redlands，CA, USA.

### 2.2. Data Repository and Database

Data sources were archived in a digital repository, while relevant and pertinent information was extracted, standardised and imported into a spreadsheet file to create the atlas database. The database was divided into three sheets, following the general approach utilised by FAO for constructing the continental atlas [47]. The first sheet includes details on the documents utilised as sources to create the atlas, the second contains the locations and the georeferencing information required for mapping, and the third encompasses the epidemiological data on the various surveys and Surra cases. Here below is a more detailed summary of the spreadsheet/database, while the column-by-column description of the three tables is provided in Supplementary file S1.

In the first sheet, a distinct numeric code (“Source_ID”) was assigned to each document, which was used to identify the data extracted from it in the other sheets. Data concerning the authors of the document (“Initials”, “Main_Author” and “Authors”), as well as its full title, country of origin, and year of publication (“Source_Name”, “Country”, “Year”) were also included. Furthermore, the type of document (“Report_type”), the presence of animal trypanosomosis information (“AT_Data”) and a note concerning whether the document is unpublished/grey literature (“Grey_document”) were included. Details such as journal, publisher, type of access (e.g., open or restricted), and external URL and availability (“External_link”, “Journal”, “Publisher”, “Access”, and “Availability”) were recorded alongside the access date and the name assigned to the file in the repository (“Accesed_on”, “File_name”).

In the second sheet, the different locations were included for each “Source_ID” in the first sheet. These locations were assigned unique identifiers (“Location_ID”) and were associated with their respective “Location_name”, “Province”, “District_island”, “Municipality”, “Geo_source” (i.e., the source of the geographical information) and “Area” (if applicable). While the exact coordinates of the surveys and Surra cases were obtained and entered into the database, they could not be included in this article for privacy reasons.

The last sheets link the various documents and locations from the previous sheets through their “Source_ID” and “Location_ID” to the results of the epidemiological surveys. Each survey was identified by a unique numeric code (“Survey_ID”). The period of sampling, including the start and end month (“Month_ST” and “Month_END”) and year (“Year_ST” and “Year_END”) was recorded, as well as the sample size (“Sample_size”). If available, the animal species sampled was indicated alongside breed, age and sex (“Species_AN”, “Breed_AN”, “Age_AN” and “Sex_AN”) and the type of farm/holding or husbandry system (“Husb_AN”). The presence or absence of *T. evansi*, the number of positive animals and prevalence (“*T_evansi*_Presence” and “*T_evansi*”) were identified. Finally, the diagnostic methods used (“Diagnostic”) were recorded, and, when applicable, the treatment given to the animals before sampling (“Chemotherapy”).

## 3. Results

Between 1997 and 2022, a total of 12,433 animal samples were analysed for *T. evansi*, and all results for the different diagnostic methods and the different animal species are summarised in Table 1. Five categories of animal species were included in these samples, with dromedary camels being the most frequently tested (63.1% of the total), followed by equids (16.4%, primarily horses and donkeys), goats (11.3%), sheep (6.7%), and cattle (2.5%). Furthermore, annual testing of dromedaries on certain islands was conducted as part of the Surra control measures implemented by the Department of Agriculture, Livestock and Fisheries of the Canary Islands.

Of the total number of samples, 61.2% were analysed with CATT/*T. evansi* method. The second most frequently used technique was the Woo method (23.9%), followed by blood smears (7%), ELISA (3.6%), PCR (3.2%), LNA (0.6%) and MIT (0.5%).

Regarding CATT/*T. evansi*, dromedaries represented the most sampled hosts (70.7%), followed by equids (13.1%), goats (9%), sheep (5.2%), and cattle (2%). Similarly, dromedaries were the most commonly tested species for PCR (67.7%), followed by sheep (11.3%), equids and goats (8.5% each) and cattle (4%). In contrast, the Woo technique was predominantly targeted at equids (33.7%), followed by dromedaries (25%), goats (23.1%), sheep (13.2%) and cattle (5%). Finally, for blood smear, ELISA, MIT and LNA tests, all samples were obtained from dromedaries.

The total number of positive samples for *T. evansi* was 601, representing 4.8% of the total number of samples tested. Given the significant variations in sensitivity among the different diagnostic tests, it is necessary to specify the number of positive samples found by each test. Specifically, for CATT/*T. evansi*, 72% of the positive samples were detected, followed by 8.65% for ELISA, 8.48% for the Woo technique, 5.6% for blood smear, 4.8% for PCR, 0.3% for LNA, and 0.17% for MIT.

Similarly, it is necessary to differentiate between each tested animal species, as the corresponding number of positive samples varies considerably. For instance, the total number of positive dromedary samples was 477 (79.5% of the total number of positives), which is considerably higher compared to the other species. The number of positive from sheep was 7.5%, followed by goats with 5.6%, equids with 4.8%, and cattle with 2.6%.

All positive samples from sheep, goats and cattle were detected by CATT/*T. evansi*. However, positive samples in equids and dromedaries were found using different techniques: for equids, 89.6% of the samples tested positive by CATT/*T. evansi*, followed by PCR with 7% and Woo technique with 3.4%. In dromedaries, the range of techniques used differed even more with CATT/*T. evansi* yielding the highest number of positives at 65.3%, followed by ELISA at 10.9%, Woo technique at 10.5%, blood smear at 7.1%, PCR at 5.6% and finally lymph node aspirate and MIT at 0.4% and 0.2%, respectively.

**Figure 1:**
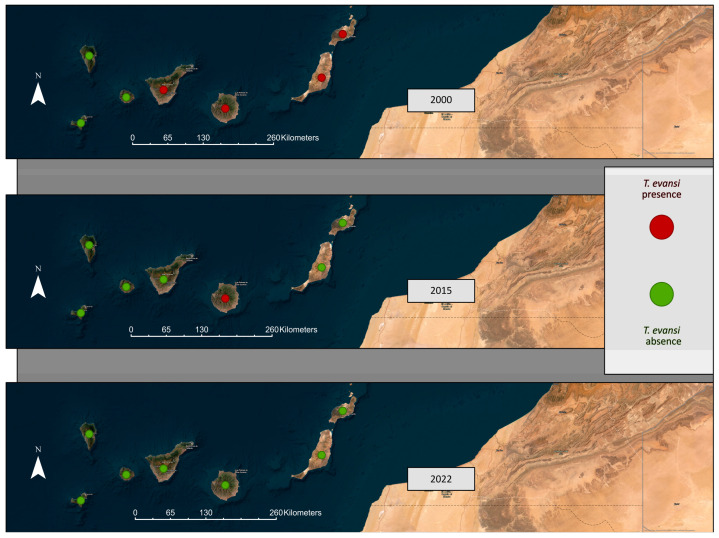
Depicts a time series representing three significant years: 2000, during which surveys and sampling were initiated in the majority of the dromedary population following the first detection in 1997; 2015, when there was an outbreak of Surra in a specific area of Gran Canaria, which prompted various epidemiological studies and formed the basis for a monitoring and control protocol established by the Government of the Canary Islands in subsequent years; and 2022, when the most recent monitoring and control data identified only three positive cases of CATT/*T. evansi* in dromedaries in the Canary Islands, with no positive results indicating the presence of this species through PCR analysis. It is important to emphasise that the classification of each island as positive relies on the confirmation of the presence of CATT/*T. evansi* positive animals.

Most of the data presented in Table 1 were collected in the context of disease control and monitoring activities (between 2015 and 2022). A total of 7709 samples were studied during this period, representing 62% of the total. The remaining 38% (4724 samples) are the result of scientific investigations carried out in the framework of academic and research activities (between 1997 and 2013) (More details in Figure 1).

**Figure 2:**
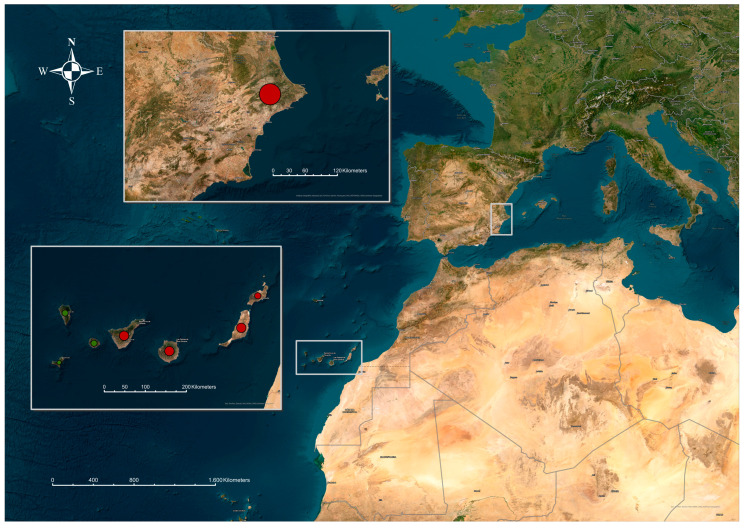
Shows presents the geographical areas in Spain where animals positive for *Trypanosoma evansi* were detected using the CATT/*T. evansi* diagnostic tool. The top region corresponds to the Iberian Peninsula, specifically the province of Alicante in the Valencian Community. In the Autonomous Community of the Canary Islands, CATT/*T. evansi* positive samples were identified on the islands of El Hierro, La Palma, La Gomera, Tenerife, Gran Canaria, Fuerteventura, and Lanzarote.

A total of 99 locations from different sources have been investigated, and the exact coordinates of each one have been obtained and included in the atlas database. The majority of these belong to the province of Las Palmas (85 locations), mainly on the islands of Gran Canaria, Fuerteventura, and Lanzarote, followed by the province of Santa Cruz de Tenerife (13 locations), mainly Tenerife Island, and the province of Alicante (1 location). Positive samples were found in 19% of the surveyed locations, with the majority being located in the province of Las Palmas (Figure 2).

### Database Completeness

A high level of completeness was achieved in this atlas. We managed to obtain a comprehensive overview of the different cases of Surra and their location over time, as well as specific information on their hosts. In most cases (approximately 90% of the records), all the required data for the atlas could be identified. For example, information on animal sampling duration, number of samples and positive cases, diagnoses, and treatment after diagnosis in a few cases, among others, has been obtained. Additionally, over 90% of the locations of the different sources have been identified at the farm level, with the remaining 10% mapped at the village/location level.

## 4. Discussion

In the atlas on animal trypanosomosis for Spain described in this paper, only studies and data on *T. evansi* were included, while atlases previously published in a few African countries focus on nagana (i.e., Sudan [48], Kenya [53], Mali [50], Zimbabwe [49], Burkina Faso [52] and Ethiopia [51]). This decision was motivated by the fact that in Spain, only the presence of this species of trypanosome has been described, apart from the previous study that revealed the presence of *T. theileri* in cattle in Spain [59]. However, it is considered as a nonpathogenic haemoparasite. Therefore, according to the results of this study and our knowledge, no other species causing animal trypanosomosis has ever been observed in Spain.

The development of this atlas will allow analysis that will lead to a better understanding of the epidemiology of Surra in the Canary Islands. As mentioned previously, the first identification of this parasite was done on the island of Gran Canaria [34]. Apart from the outbreak diagnosed and eradicated in the province of Alicante, which originated from the exportation of apparently healthy dromedaries carrying the disease from the Canary Islands [17,41], the Canary Islands is the only region in Spain that has consistently reported cases of Surra throughout the study period. Similarly, it is noted that most cases of the disease have been concentrated in the eastern part of the Canary Islands.

Therefore, after collecting a large number of samples throughout various studies, we can conclude that among all potential hosts present in Spain, the dromedary, historically with a large population in the Canary Islands, is the main host of *T. evansi*, with cases appearing in other species being derived from those already present in them.

However, several limitations can be found in this atlas. For example, despite obtaining various specific locations (mainly camel farms) where investigations for the parasite have been conducted and positive animals have been found, the maps created and included in this document present generalised locations, mainly due to privacy reasons.

In addition, the exact sampling location has not always been available in the specific documents analysed. In these cases, solely island-level inclusion of the farm’s location was feasible within the atlas database. This allows us to highlight the importance of knowing and documenting specific locations in future studies, with the aim of having the maximum possible information in this regard.

Sampling has predominantly focused on dromedaries, one of the various species that could potentially serve as hosts for *T. evansi*, given their high vulnerability [20] and the occurrence of Surra cases in the Canary Islands. However, although studies with other possible hosts have been mainly conducted in academic circles [44,60], it is imperative to carry out exhaustive research in other species (especially those from areas close to dromedary populations or regions with historical records of positive animals) to obtain a representative sample that aligns with the studied dromedaries.

Regarding the diagnostic samples used, the most utilised method to detect animals positive for Surra has been CATT/*T. evansi*. This diagnostic method primarily detects a specific antigen of *T. evansi*, specifically the A variant [7,61]. However, the literature describes the existence of a B variant that lacks this specific antigen, raising controversy over the use of CATT/*T. evansi* as the primary detection method [62]. In some studies, PCR was subsequently performed to confirm the presence of the parasite in animals that tested positive with this assay. However, without complementary tests such as parasitological techniques (Woo technique, blood smear examination, etc.), there is a possibility that the B variant may be present. This should be considered and investigated in future studies to ensure an accurate determination of the presence or absence of *T. evansi* in the samples. The correct detection of these pathogenic trypanosomes is becoming increasingly important, as despite not being currently classified as a zoonosis due to its asymptomatic nature, several studies suggest that it could potentially be considered as one [32,63]. Another important limitation to consider is the absence of sample collection and entomological studies. Due to the mechanical nature of *T. evansi* transmission, it is crucial to identify various vectors responsible for sustaining and propagating the disease [36]. Only Rodriguez et al. [43] reported the presence of *Stomoxys calcitrans* as a potential primary vector in Surra transmission in the Canary Islands. However, this hypothesis remains unverified due to the absence of genetic tests for confirmation.

## 5. Conclusions

Given the lack of a comprehensive database on the occurrence of Surra and other pathogenic trypanosomes in Spain, this study represents a milestone in understanding the disease and its distribution in space and time. Not only does it provide insight into the events to date, but it also serves as a tool for disease control in the future.

It is necessary to continue obtaining more data from animals and new entomological samplings in order to achieve a better understanding of the disease in Spain and the Canary Islands. By using new data and publications from these future investigations, the atlas of trypanosomosis in Spain will be continually updated and improved. The upcoming inclusion of entomological study results from different islands in the Canary Archipelago, where Surra has been present in the past, is a good example of the usefulness of the atlas as a tool for disease control and monitoring. Additionally, this atlas will allow us to target a comprehensive study of trypanosomosis in other animal species, different from the dromedary, that are susceptible to Surra, aiming to fill in the current information gaps. This atlas intends to be a useful tool for the official animal health authorities, primarily in the Canary Islands, to improve monitoring and control activities, mainly through risk analysis and consideration of at-risk herds on the different islands. Furthermore, in the case of new disease outbreaks, this atlas can provide information and guidance for implementing proper prophylaxis and control measures in affected regions of Spain.

## Figures and Tables

**Table 1:** Number of positive cases and prevalence for *T. evansi* in different species and diagnostic methods in Spain between 1997–2022.

Diagnostic Method	Animal Species	Animal Samples (n)	Number of Infections (n)/Prevalence (%)
			*T. evansi*
CATT/*T. evansi*	Dromedary	5385	311 (5.77%)
	Equids	1001	26 (2.59%)
	Cattle	148	16 (10.81%)
	Goat	687	34 (4.95%)
	Sheep	393	45 (11.45%)
	Subtotal	7614	432 (5.67%)
Woo technique	Dromedary	741	50 (6.75%)
	Equids	1001	1 (0.09%)
	Cattle	148	0 (%)
	Goat	687	0 (%)
	Sheep	393	0 (%)
	Subtotal	2970	51 (1.72%)
Blood smear	Dromedary	876	34 (3.88%)
ELISA	Dromedary	444	52 (11.71%)
Lymph node aspiration	Dromedary	73	2 (2.74%)
MIT	Dromedary	56	1 (1.78%)
PCR	Dromedary	271	27 (9.96%)
	Equids	34	2 (5.88%)
	Cattle	16	0 (0%)
	Goat	34	0 (0%)
	Sheep	45	0 (0%)
	Subtotal	400	29 (7.25%)

(MIT) Mice inoculation test; (CATT/*T. evansi*) Card agglutination test; (PCR) Polymerase chain reaction; (ELISA) Enzyme-linked immunosorbent assay; (n) Number.

## Data Availability

Most of the dataset supporting the conclusions of this article is included within the article. The datasets not included in this study are available upon request from the corresponding author.

## Supplementary Materials

The following supporting information can be downloaded at: https://www.mdpi.com/article/10.3390/ani14020243/s1, File S1: Structure of the database.
