# Supplementary material for: An Atlas of Surra in Spain: A Tool to Support Epidemiological Investigations and Disease Control
Source: Animals (Basel). 2024 Jan 12;14(2):243. doi: 10.3390/ani14020243 (PMC10812746; doi:10.3390/ani14020243)
Supplement: Supplementary file 1 [file animals-14-00243-s001.zip › animals-2792210-supplementary.pdf]

Supplementary Material:

File 1: Structure of the database.

Source sheet

- **Source_ID**: Unique numeric identifier for each document used in the database.
- **Initials**: Initial of the name of the main author of the document.
- **Main_Author**: Surname of the main author of the document.
- **Authors**: Surname and initial of the name of all the authors of the document.
- **Source_Name**: Full title of the document.
- **Country**: Country where the document was made.
- **Year**: Year in which the article was published.
- **Grey_document**: This designation includes documents from sampling and/or unpublished studies.
- **AT_Data**: Presence of information on animal trypanosomosis in the document.
- **File_name**: Name of the file in the repository.
- **External_link**: Link to access online publication.
- **Additional_information**: Additional notes relating to each of the documents.
- **Journal**: Scientific journal in which the paper has been published.
- **Publisher**: Editor of the scientific journal.
- **Access**: Shows via which platform the document can be accessed.
- **Availability**: Indicates whether the document is available for retrieval from the internet.
- **Accesed_on**: Date on which the document was obtained.
- **Report_type**: Document type.
- **Conference**: In the case of oral communications, conference where the presentation took place.

Geo_data sheet

- **Source_ID**: Numeric identifier of the document from which the localization originates (related to the same section in the Source sheet).
- **Location_ID**: Numeric identifier of the specific location.
- **Country**: Country to which the location belongs.
- **Location_name**: Name assigned to the specific location or area.
- **Province**: Province to which the location belongs.
- **District_Island**: In the case of the Canary Islands, the island to which the location belongs.
- **Municipality**: Municipality to which the location belongs.
- **Long:** Longitude
- **Lat:** Latitude
- **Geo_source**: Application or website used to obtain and/or geo-reference the location.
- **Location_notas**: Notes relating to each of the locations.
- **Area**: In some cases, where it has not been possible to obtain an exact location, total area in km^2 where the farm is estimated to be located.

Epi_data sheet

- **Source_ID**: Numeric identifier of the document from which the sample is taken (related to the same section in Source sheet and Geo_data sheet).
- **Location_ID**: Numerical identifier of the location, relating the sampling to the location.
- **Survey_ID**: Numerical identifier of the sample.
- **Month_ST**: Month in which sampling started.
- **Year_ST**: Year in which sampling started.
- **Month_END**: Month in which sampling ended.
- **Year_END**: Year in which sampling ended.
- **Sample_size**: Total number of animals sampled.
- **Note_size**: Comments on the number of animals sampled.
- **Species_AN**: Species sampled.
- **Breed_AN**: Specific breed of animals sampled.
- **Age_AN**: Age or age range of animals sampled.
- **Sex_AN**: Sex of animals sampled.
- **Husb_AN**: Type of livestock where the animals were kept.
- **T_evansi_presence**: Presence or absence of *T. evansi* in animals.
- **T_lewisi_presence**: Presence or absence of *T. lewisi* in animals.
- **T_evansi**: Number of animals infected with *T. evansi.*
- **T_lewisi**: Number of animals infected with *T. lewisi*.
- **Diagnostic**: Extract from the document on the diagnostic technique used.
- **Diagnostic_CAT**: Full name of the diagnostic technique.
- **Diagnostic_CAT_2**: Acronym/acronym of the diagnostic technique.
- **Chemotherapy**: Indication of treatment to the animals.
- **Chemo_CAT**: Name of the medical product used.
- **Notes**: Sampling notes.
- **Additional_info**: Additional information on sampling.
